# Assessment of Quality of Life After Ventral Hernia Repair: A Prospective Observational Study at a Tertiary Care Centre

**DOI:** 10.7759/cureus.26136

**Published:** 2022-06-20

**Authors:** Mahendra Lodha, Darshan Patel, Mayank Badkur, Satya Prakash Meena, Ashok Puranik, Ramkaran Chaudhary, Indra Singh Choudhary, Metlapalli V Sairam, Anupam Singh Chauhan, Rashi Lodha

**Affiliations:** 1 General Surgery, All India Institute of Medical Sciences, Jodhpur, Jodhpur, IND; 2 Medical Intern, Dr. Sampurnanand Medical College, Jodhpur, IND

**Keywords:** short-term outcome, open ventral hernia repair, quality of life (qol), laproscopic ventral hernia repair, abdomen ventral hernia

## Abstract

Background

Ventral hernias are usually repaired by an open or laparoscopic approach. Quality of life after ventral hernia repair is a very important but often underestimated parameter. This prospective observational study was conducted to assess the quality of life and other related parameters after all types of ventral hernia repair, mainly between open and laparoscopic repairs.

Objectives

This study aimed to determine the quality of life after ventral hernia repairs. We also analysed and compared various parameters such as outcomes and satisfaction, postoperative pain, and complications between laparoscopic and open ventral hernia repair.

Methods

This was a hospital-based prospective observational study conducted from January 2020 to December 2021, which included a total of 70 patients with ventral hernias. Thirty-nine patients underwent open repair and 31 patients underwent laparoscopic repair. Demographic data and other data such as postoperative hospital stay, return to activity, postoperative pain, complications, and quality of life were collected and analysed.

Results

The distribution of different types of hernias observed in our study included 34% incisional hernias, 33% umbilical and paraumbilical hernias, and 33% epigastric hernias. The incidence of complications was significantly less in laparoscopic repair compared to open repair. Also, satisfaction at 1 month was significantly more in the laparoscopic group compared to the open group. However, there is no significant difference in the postoperative pain, postoperative hospital stay, return to activity, satisfaction at discharge, and quality of life at 1 month in both the laparoscopic and open repairs.

Conclusion

Laparoscopic ventral hernia repairs are associated with lesser complications and higher satisfaction. The use of tackers and trans-fascial sutures can significantly increase postoperative pain in laparoscopic repair and is the major factor affecting the short-term quality of life in laparoscopic repairs. As there is no difference in postoperative pain, hospital stay, and return to activity, laparoscopic repairs should be preferred wherever possible in view of fewer complications and higher satisfaction.

## Introduction

Ventral hernia of the abdomen is defined as a protrusion of the abdominal viscera through a non-hiatal, non-inguinal defect in the fascia of the abdominal wall. They are commonly seen in clinical practice. Patients usually present with swelling or bulge over the abdomen, which is usually reducible on lying down. Sometimes it may be associated with dull aching pain [1].

Ventral hernias include umbilical, epigastric, Spigelian, lumbar, and incisional herniae [1]. According to the European Hernia Society, ventral hernias are classified as primary ventral hernia and incisional hernia. A primary ventral hernia occurs over the previously normal skin while an incisional hernia occurs over the incision of a previously operated site. They are further divided based on the length and width of the defect size [2].

Microscopic tissue tears secondary to repetitive stresses are responsible for the pathogenesis of ventral hernia. The main factors responsible for it are chronic cough, urinary straining, constipation, pregnancy, and obesity amongst many others [3].

Ventral hernias are usually repaired by an open or laparoscopic approach. Recently many surgeons prefer a hybrid approach, mainly for incisional hernia repair [4]. Laparoscopic hernia repairs are usually associated with less postoperative pain, wound infections, and significantly improved quality of life in the long term [5].

Numerous research has already been done comparing the long-term quality of life before and after individual umbilical, incisional, and other subtypes of ventral hernias [6-14]. However, there is very minimal research material available comparing the short-term quality of life after all types of ventral hernia repairs. Also, there is very little data available for short-term quality of life after ventral hernia repair in India. 

Hence, this prospective observational study is conducted to assess the quality of life after all types of ventral hernia repair, be it open or laparoscopic, small or large, repaired primarily or with mesh hernioplasty. 

How does this research fit within the existing literature?

There were a few studies about the quality of life among patients undergoing ventral hernia repair. However, there is little literature regarding the same from India. So we conducted a study comparing the quality of life among patients undergoing open and laparoscopic repair in the Indian subcontinent.

## Materials and methods

Research question

Among patients undergoing ventral hernia repair, what is the quality of life postoperatively among those undergoing open repair as compared to those undergoing laparoscopic repair?

Aim 

The aim of the study was to assess the quality of life at 1 month postoperatively after ventral hernia repair.

Objective

The study has the following objectives: 1. To compare the outcome and satisfaction among the patients who are treated by open versus laparoscopic ventral hernia repair; 2. To measure and compare the quality of life (QOL) by QOL assessment scale at 1 month postoperatively in patients operated with open and laparoscopic ventral hernia repair; 3. To assess and compare postoperative pain in patients operated with open and laparoscopic ventral hernia repair; 4. To assess and compare postoperative complications like wound infections, seroma, hematoma, and mesh infection in patients operated with open and laparoscopic ventral hernia repair; and 5. To assess and compare the quality of life at 1 month after large (>7 cm) and small (<7 cm) ventral hernia repairs. 

Study setting

This study was conducted on patients operated for ventral hernia at the All India Institute of Medical Sciences, Jodhpur, a tertiary care hospital in Rajasthan, India. 

Study design 

This was a hospital-based prospective observational study.

Study duration 

After approval by the Institutional Ethics Committee, the study was conducted from January 2020 to December 2021. 

Sampling 

The time-bound study included cases from January 2020 to December 2021 after applying inclusion and exclusion criteria. We were expecting a sample size of 100 over the period of 2 years, but due to the shut-down of elective operations during the COVID-19 pandemic, we could not complete the sample size. A total of 70 patients met the inclusion criteria and were included in this study. 

Study participants 

Inclusion Criteria 

Patients (>18 years of age) who presented with ventral hernias and were operated on electively in our hospital were included after obtaining written consent. 

Exclusion Criteria

The exclusion criteria were: 1. Patients who presented to acute surgical care unit in view of surgical emergencies like acute intestinal obstruction, 2. Lumbar hernias, and 3. Mentally disabled patients.

Data collection

This was a single-center prospective observational study conducted at AIIMS Jodhpur. Patients who presented with ventral hernias between 1 January 2020 to 31 December 2021, and who met the inclusion criteria, were included. The study was approved by the Ethical Committee of AIIMS Jodhpur, Rajasthan, India (approval number AIIMS/IEC/2019-20/1002). All procedures followed were in accordance with the ethical standards of the responsible committee on human experiments (institutional and national). Informed consent was obtained from all patients being included in the study. 

For the proper record, a proforma is prepared for the study. All patients involved in the study underwent a detailed clinical examination and a detailed history according to the designed proforma. The demographic data of the patient, risk factors, comorbidities, previous surgical history, investigations, type, and size of the hernia, defect size, and the content of the hernia was collected preoperatively. All patients were explained about surgical options available and were given the freedom to choose the surgery they want to undergo. Patients then underwent surgery by laparoscopic or open technique. Intraoperative data such as findings, the procedure performed, drain placement, complications, and need for conversion, were also noted. Postoperative data such as pain on day 1 and on discharge, wound complications, other systemic complications, drain removal day, postoperative ICU care requirement, postoperative hospital stay, and condition at discharge was also collected for each patient. Postoperative pain is a significant factor affecting the immediate quality of life after ventral hernia repair. For postoperative pain score measurement, we used the visual analog scale (VAS). The patient was asked to quantify the pain on a scale of 1 to 10. The more the number, the more severe is the pain. We took three serial VAS scores at the intervals of 12 hours each and calculated the average pain score from them. We also calculated the number of analgesic dosages required for each patient in the postoperative period. 

For determining the outcome, we measured two parameters: average hospital stay (in days) and average time to return to activity (in days). Return to activity was considered when the patient was completely ambulated and performing routine activities without any pain. Both of these variables were compared between laparoscopic and open ventral hernia repairs.

For measuring satisfaction, we asked the patient to quantify the feeling of satisfaction after the surgery, on a scale of 1 to 10, where 1 is ‘very unsatisfied’ and 10 is ‘highly satisfied’. Mean satisfaction scores at discharge and at 1 month were calculated and compared between laparoscopic and open groups.

We also assessed the patients for postoperative complications after 1 month of the surgery. The wound was assessed for any seroma, hematoma, or infection. Also, complications like continuous pain at 1 month, postoperative ileus, and the need for a re-laparoscopy were assessed. The incidence of complications was calculated and compared between laparoscopic and open surgeries. The drain was removed when the collection was less than 30 ml per day. Patients were discharged with stable vitals.

After discharge, patients were assessed for quality of life at 30 days postoperatively by the self-made quality of life assessment questionnaire, consisting of 12 questions. The questions were related to the physical, emotional, and social well-being of the patient.

Each question was given a score of 1 to 5, 1 being ‘strongly disagree’ and 5 being ‘strongly agree’. The total score of all questions was calculated. The lower the score, the better the quality of life. The mean QOL score at the first month was calculated and compared for laparoscopic and open ventral hernia repairs. The mean QOL score of large and small ventral hernias was also calculated and compared between these two groups.

All data were entered in a patient proforma by the resident in the ward and finally compiled in a Microsoft Excel sheet (Microsoft Corporation, Redmond, USA). 

Statistical analysis

Data were entered and analyzed using SPSS Statistics version 28 (IBM Corp., Armonk, USA). Nominal data were described using frequency or percentages and compared using the chi-square test or Fischer's exact test. Ordinal data were described using median and interquartile range (IQR) and compared using the Mann-Whitney U test. Continuous data were described using mean +/- SD and compared using an unpaired t-test. A p-value of <0.05 was considered statistically significant.

Ethical consideration

Ethical clearance was obtained from the Institutional Ethical Committee of AIIMS Jodhpur (approval number AIIMS/IEC/2019-20/1002). Patients were enrolled after taking informed consent. Patient details were kept confidential. No compromise in patient care was done.

## Results

Seventy patients were included in this study: 39 patients underwent open ventral hernia repair and 31 patients underwent laparoscopic ventral hernia repair. Quality of life was compared in both the groups along with other outcomes including postoperative pain, analgesic usage, postoperative hospital stay, return to activity, postoperative complications, and satisfaction. We also compared the quality of life between large and small ventral hernia repairs. 

The mean age of the male patients was 52.08 +/- 13.88 and of the female patients was 50.2 +/- 11.00. A total of 35 male patients and 35 female patients participated in the study. The gender distribution in the study was found to be comparable. The co-morbidities associated with these patients included diabetes mellitus, hypertension, coronary artery disease, and asthma and they were not distributed evenly. Fifty-five patients had no major risk factor for a hernia. However, eight patients had constipation, four patients had a chronic cough and four patients had difficulty in micturition. Out of 70 patients, 63 patients had small hernias and seven patients had large hernias. The number of patients with large ventral hernias was very less compared to small hernia patients. Thirty-nine patients underwent open ventral hernia repair and 31 patients underwent laparoscopic ventral hernia repair. Out of 70, 15 patients required abdominal drain while 55 patients did not require any drain (Table 1).

**Table 1:** Distribution of different variables in the study

	Variables	No. of patients
Age (years)	Male: 52.08 +/- 13.88	35
	Female: 50.2 +/- 11.00	35
Gender	Male	35
	Female	35
Comorbidities	Hypertension	15
	Diabetes	10
	Ischemic heart disease	3
	Asthma	4
Defect size	Small (<7)	63
	Large (≥7)	7
Choice of treatment	Open	39
	Laparoscopic	31
Drain placement	With drain	15

Out of 70, 23 patients had epigastric hernia, 23 patients had umbilical and paraumbilical hernias, and 24 patients had incisional hernias. The distribution of different types of hernias was comparable. Out of 36 patients who started as laparoscopic repairs, five procedures were converted to open repairs. The major reason for conversion was the presence of dense adhesions in four patients and because of irreducible content in one patient (Table 2). 

**Table 2:** Number of laparoscopic repairs converted to open surgery and reasons

Conversion to open	No. of patients		Reason for conversion
Yes	5	4	Dense adhesions
		1	Irreducible content
No	31		

**Figure 1:**
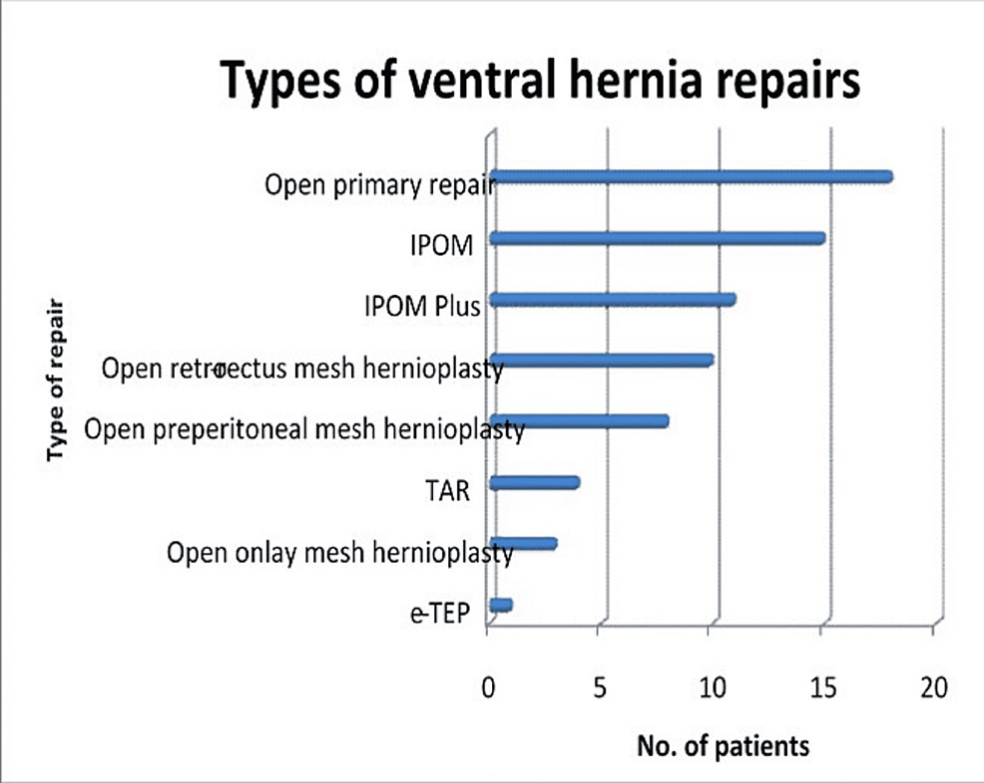
Types of ventral hernia repairs IPOM: intra-peritoneal onlay mesh, TAR" transversus abdominis muscle release, e-TEP: extended totally extraperitoneal repair

The most common repair done was open primary repair in 18 patients (for small ventral hernias with defect <2 cm), followed by IPOM (Intra-Peritoneal Onlay Mesh) in 15 patients, followed by IPOM Plus in 11 patients, open retro-rectus mesh hernioplasty in 10 patients, and open preperitoneal mesh hernioplasty in eight patients, TAR (Transversus Abdominis muscle Release) in four patients, open onlay mesh hernioplasty in three patients, and e-TEP(Extended Totally Extra-Peritoneal repair)** **in one patient (Figure 1). 

The p-value for comparison between the QOL at the first month in open ventral hernia repair (QOL 23.8 +/- 11.9) and laparoscopic ventral hernia repair (QOL 20.7 +/- 7.33) is 0.187, which means that there is no significant difference (Table 3).

**Table 3:** Comparison of quality of life (QOL) at first month between open and laparoscopic ventral hernia repairs

Variables		No. of patients	Mean QOL score	SD	t-value	p-value (Independent t-test)
Choice of treatment	Open	39	23.8	11.9	1.332	0.187
	Laparoscopic	31	20.7	7.33		

We have analysed the outcome by considering two parameters: average hospital stay (in days) and return to activity (in days). It is observed that there is no significant difference in the average postoperative hospital stay between open (4.12 +/- 6.38 days) and laparoscopic (2.54 +/- 1.31 days) ventral hernia repair with a p-value of 0.18. Also, there is no significant difference in return to activity between open (3.07 +/- 1.89 days) and laparoscopic (3.09 +/- 1.68) with a p-value of 0.963 (Table 4). 

**Table 4:** Comparision of outcomes between open and laparoscopic ventral hernia repairs

Variables	Open (Mean±SD)	Laparoscopic (Mean±SD)	p-value (Mann-Whitney U test)
Hospital stay	4.12±6.38	2.54±1.31	0.18
Return to activity	3.07±1.89	3.09±1.68	0.963

There is no significant difference in the satisfaction score at discharge between open (8.25 +/- 1.27) and laparoscopic (8.45 +/- 0.96) ventral hernia repair with a p-value of 0.685. However, there is a significant difference in the satisfaction score in first month between open (7.18 +/- 2.20) and laparoscopic (8.80 +/- 1.40) ventral hernia repair with a p-value of 0.007 (Table 5). Laparoscopic hernia repairs are associated with higher satisfaction scores compared to open hernia repairs. 

**Table 5:** Comparision of satisfaction between open and laparoscopic ventral hernia repairs

Variables	Open (Mean±SD)	Laparoscopic (Mean±SD)	p-value (Mann-Whitney U test)
Satisfaction score at discharge	8.25±1.27	8.45±0.96	0.685
Satisfaction score at 1^st^ month	7.18±2.20	8.80±1.40	0.007

There is no significant difference in the postoperative pain between open (5.49 +/- 2.24) and laparoscopic ventral hernia repair (6.03 +/- 1.99) with a p-value of 0.292. Also, there is no significant difference in the average analgesic dose usage between open (2.15 +/- 2.97) and laparoscopic ventral hernia repair (6.06 +/- 3.07) with a p-value of 0.216 (Table 6). However, laparoscopic repairs are associated with a higher number of analgesic doses requirement compared to open repairs.

**Table 6:** Comparision of postoperative pain (VAS score) and analgesic usage (average number of doses) between open and laparoscopic ventral hernia repairs VAS: visual analog scale

Variables	Open (Mean±SD)	Laparoscopic (Mean±SD)	p-value (Mann-Whitney U test)
Average pain score	5.49±2.24	6.03±1.99	0.292
Analgesic usage	2.15±2.97	6.06±3.07	0.216

Out of 39 patients who underwent open repairs, 17 patients developed any of the defined complications. Whereas out of 31 patients who underwent laparoscopic repairs, only three patients developed any of the defined complications. According to this data, it is observed that there is a significant difference in the incidence of complications between open and laparoscopic ventral hernia repair with a p-value of 0.001 (Table 7). 

**Table 7:** Comparision of incidence of complications between open and laparoscopic ventral hernia repairs

Complications	Choice of treatment		p-value
	Open	Laparoscopic	0.001
Yes	17	3	
No	22	28	

There is a significant difference in the quality of life at the first month between large (QOL 34.6 +/- 11.9) and small ventral hernia repair (QOL 21.1 +/- 9.12) with a p-value of 0.027 (Table 8). Small ventral hernia repairs are associated with a better quality of life compared to large ventral hernia repairs. 

**Table 8:** Comparision of quality of life (QOL) at first month between large and small ventral hernia repairs

Variables		No. of patients	Mean QOL score	SD	t-value	p-value (independent t-test)
Defect size	Small (<7)	63	21.1	9.12	2.906	0.027
	Large (≥7)	7	34.6	11.9		

## Discussion

Any surgery over the abdomen can have serious consequences on the physical, social and mental well-being of the patient and can hamper the quality of life, at least for the first few weeks after the surgery. Our primary aim was to assess and compare the quality of life in ventral hernia repairs. We also compared other outcomes like postoperative pain, complications, average hospital stay, and time to return to activity. Alongside we have also assessed various types of ventral hernias encountered, types of various procedures performed, common risk factors, and comorbidities. 

Quality of life is different from the health status of the patient. Quality of life focuses on the subjective appraisal of a patient’s well-being whereas health status is an objective measure. We have used 12 questionnaire proforma which have questions covering three major aspects affecting the patient’s wellbeing, including physical health, mental health, and social health. The physical health domain includes items on mobility, daily activities, energy, and pain. The mental health domain includes self-image, mental status, and tension. The social domain includes social support, outdoor activity, work performance, sexual life, and personal relationships. Guzman-Pruneda et al. conducted a retrospective cohort study to a comparison of quality of life between open and robotic ventral hernia repair. They used HerQLes for QOL measurement, which also has the same three domains as our study [13].

This prospective observational study conducted in the Department of General Surgery, All India Institute of Medical Sciences, Jodhpur, included a total of 70 patients. Out of which, 39 patients underwent open ventral hernia repair and 31 patients underwent laparoscopic ventral hernia repair. Quality of life and other outcomes were compared in both groups. We also compared the quality of life between large and small ventral hernia repairs. 

A total of 35 male patients and 35 female patients participated in the study. The gender distribution in the study was found to be comparable. The mean age of the male patients was 52.08 +/- 13.88 years and of the female patients was 50.2 +/- 11.00 years. The comorbidities associated with these patients included diabetes mellitus, hypertension, coronary artery disease, and asthma and they were not distributed evenly. Fifty-five patients had no major risk factor for a hernia. However, eight patients had constipation, four patients had a chronic cough and four patients had difficulty in micturition. Out of 70 patients, 63 patients had small hernias and seven patients had large hernias. The number of patients with large ventral hernias was very less compared to small hernia patients. Out of 70, 15 patients required abdominal drain while 55 patients did not require any drain. 

The common types of ventral hernias encountered in our study were epigastric (33%), umbilical and para-umbilical (33%), and incisional (34%). These three common types of hernias were almost equal in this study. This finding is comparable to the study done by Rutkow et al. where they observed a similar demographic distribution. In their study, one-third of the patients had incisional hernias and two-thirds of the patients had primary ventral hernias [15].

There are a number of procedures available for ventral hernia repairs. In our study, the most common repair done was open primary repair in 18 patients (for small ventral hernias with defects <2 cm). Open primary repair was the most common procedure because the majority of the epigastric hernias were <2 cm in size and open primary repair is the treatment of choice for hernias <2 cm in size. IPOM and IPOM Plus were the second and third common repairs performed, respectively. Because the majority of the hernias had defect size of 2-7 cm and laparoscopic repair is the treatment of choice for medium-size hernias. Also, it was preferred by the majority of the patients, compared to long incisions over the abdomen. Four patients underwent TAR because of large defect sizes (>7 cm). e-TEP is still in the experimental phase and so only one patient underwent that particular procedure. This was a non-randomized observational study, so patients were explained the different surgical options available and were given the freedom to choose the treatment after considering all pros and cons. 

Out of 36 patients who started with laparoscopic repairs, five procedures were converted to open repairs. The presence of dense adhesions was the major reason for conversion (in four patients). One patient required conversion because of the irreducible content and risk of bowel injury. LeBlanc et al. had observed a conversion rate of 3.6% and the major reason was secondary to enterotomies in 1.8%. This finding is not comparable to our study as we had zero incidences of intraoperative enterotomy. This may be because of the very small sample size in our study [16]. However, the overall conversion rate (13.9%) was higher compared to the above study. We kept our threshold low for conversion in case of dense intra-peritoneal adhesions. 

We have observed that there is no significant difference in the average postoperative hospital stay between open and laparoscopic ventral hernia repair with p-value of 0.18. Because the majority of the small and medium-size hernia patients who underwent open repairs had some sort of pain secondary to long abdominal incisions. In contrast, laparoscopic hernia repairs had no major incisions over the abdomen still because of the usage of tackers and trans-fascial sutures intraoperatively, patients experienced moderate to severe pain in the postoperative period and that leads to an overall same hospital stay as open repairs. Al Chalabi et al. found that there is no significant difference in the length of postoperative hospital stay between laparoscopic versus open ventral hernia repairs [17]. A randomized control trial by Shankaran et al. was also suggestive of higher postoperative pain with the use of intraoperative tackers [18]. Although not significant, there is some difference in postoperative hospital stay between laparoscopic and open repairs. Laparoscopic repairs have ~1.5 days’ early discharge compared to open repairs. This can be attributed to the higher postoperative complications associated with open repairs, leading to increased hospital stay compared to laparoscopic repair. Akinci et al. also proved that postoperative complications are associated with a significantly higher postoperative hospital stay [19].

There is no significant difference in return to activity between open and laparoscopic repairs. The average duration for return to activity in both groups was 3 days. Although, patients were encouraged to ambulate from postoperative day 1, because of the associated pain in both laparoscopic and open repairs, return to activity took almost 3 days in both the groups. A study done by Forbes et al. was also comparable to the above finding. They have also observed that postoperative pain is associated with a significantly more time to return to activity, leading to decreased short-term quality of life [20].

Patient satisfaction is often underestimated component of patient care. But it is the major factor affecting the overall patient perception. In our study, it was found that there is no significant difference in the satisfaction score at discharge between open and laparoscopic ventral hernia repairs. Because at the time of discharge, the cosmetic benefit of the laparoscopic surgery was outweighed by pain secondary to tackers and that affected the satisfaction score significantly at the time of discharge. A study by Eriksen et al. was also suggestive of very high dissatisfaction after laparoscopic ventral hernia repairs secondary to higher pain associated with it [21]. However, there is a significant difference in the satisfaction score in 1st month between open and laparoscopic ventral hernia repair. Laparoscopic hernia repairs are associated with more satisfaction compared to open hernia repairs. Because at 1 month, pain in both the groups was minimal but laparoscopic repairs have the advantage of better cosmesis and that could be the main reason for increased patient satisfaction at 1 month. Chronic pain and complications are major predictors of long-term satisfaction after laparoscopic ventral hernia repairs according to Langbach et al. [22]. In our study, because of the very less incidence of continuous pain at 1 month, satisfaction scores were improved significantly at 1 month compared to open repairs.

Postoperative pain is a significant determinant of the overall immediate quality of life. In our study, there is no significant difference in the postoperative pain between open and laparoscopic ventral hernia repair. However, there is a non-significantly higher pain in laparoscopic repair. We attributed this finding to the use of tackers and trans-fascial sutures in laparoscopic repairs. A randomized control trial by Shankaran et al.was also suggestive of higher postoperative pain with the use of intraoperative tackers [18]. Also, there is no significant difference in the average analgesic dose usage between open and laparoscopic ventral hernia repair. However again, laparoscopic repairs are associated with a three times higher number of analgesic doses requirement compared to open repairs. 

While assessing and comparing complications, it was found that out of 39 patients who underwent open repairs, 17 patients developed any of the defined complications. Whereas out of 31 patients who underwent laparoscopic repairs, only three patients developed any of the defined complications. According to the above data, it is observed that there is a significant difference in the incidence of complications between open and laparoscopic ventral hernia repair with a p-value of 0.001. This finding is similar to the study by Davies et al. in which laparoscopic repairs were associated with significantly lower complications compared to open repairs [23]. 

There is a significant difference in the quality of life in the first month between large and small ventral hernia repair. Small ventral hernia repairs are associated with a better quality of life compared to large ventral hernia repairs. Large hernia repairs were done by open approach in the majority of the patients and were usually associated with extensive dissection in the abdominal wall. This leads to significantly higher pain, more complications, and bad cosmesis, leading to poor QOL in large hernia repairs compared to small hernia repairs. 

When the quality of life at first month was compared between open and laparoscopic repairs, it was observed that there is no statistically significant difference between the two groups. Based on the above discussion, it is evident that open repairs were associated with significantly more complications, more postoperative hospital stay, and decreased satisfaction at 1 month, leading to the decreased overall quality of life of these patients. We believe that the quality of life of patients with the laparoscopic repair was also negatively affected by the pain due to the usage of tackers, leading to overall similar quality of life compared to open repairs. Our findings are similar to a meta-analysis conducted by Al Chalabi et al. which was suggestive of comparable quality of life and outcomes between laparoscopic versus open ventral hernia repairs [17]. A study by Colavita et al. was also suggestive of comparable long-term quality of life between open and laparoscopic ventral hernia repairs. However, short-term quality of life at 1 month was lower in laparoscopic repairs than in open repairs secondary to more pain [24]. In our study, because of minimal pain at first month in the majority of the laparoscopic repair patients, short-term quality of life is comparable to open repairs. 

The strength of our study is that there are very few previous publications that have studied and compared the short-term quality of life along with all the associated parameters like postoperative pain, hospital stay, return to activity, complications, and satisfaction between laparoscopic and open repairs. 

The limitations of our study include small sample size, non-random nature of the study, use of self-made QOL tool, and subjective variation in terms of individual patient's perception. A multi-center, randomized trial with a larger sample size, with the use of a properly validated QOL tool, would help to overcome these shortcomings. 

This study was carried out during the times of the COVID-19 pandemic. Despite the odds and difficulties of elective operations, we could operate and gather the data for a maximum number of ventral hernia cases.

## Conclusions

Laparoscopic ventral hernia repairs are associated with lesser complications and higher satisfaction. The use of tackers and trans-fascial sutures can significantly increase postoperative pain in laparoscopic repair and is the major factor affecting the short-term quality of life in laparoscopic repairs. There is no difference in postoperative pain, hospital stays, and time to return to normal activity. Laparoscopic hernia repairs are preferred to open repairs wherever feasible in view of lower complications, and higher satisfaction scores.
